# N-terminal proteolytic processing of G proteiin-coupled receptor 37 (GPR37)

**DOI:** 10.1186/2193-1801-4-S1-P26

**Published:** 2015-06-12

**Authors:** Orvokki Mattila, Ulla Petäjä-Repo

**Affiliations:** University of Oulu, Finland

**Keywords:** GPR37, AR-JP, shedding

GPR37 is a G protein-coupled receptor that is abundantly expressed in the brain and has been implicated in dopaminergic signaling [1]. The receptor has been identified as a substrate of the ubiquitin ligase parkin and it has been linked to the autosomal recessive juvenile parkinsonism (AR-JP), an early onset familial Parkinson’s disease. The loss of parkin function and deficits in the ubiquitin proteasome pathway were proposed to cause intracellular accumulation of unfolded GPR37 leading to the AR-JP pathogenesis. [2] Here, we found that while GPR37 appears to mature normally in a heterologous expression system, the receptor is subject to proteolytic cleavage at its large N-terminal extracellular region. To study this proteolytic processing, we used stably and transiently transfected human embryonic kidney (HEK) 293 and SH-SY5Y neuroblastoma cells that express N- and C-terminally epitope-tagged human GPR37. N-terminal sequencing of the cleaved C-terminal receptor fragment revealed that GPR37 is cleaved between Glu187and Gln188 and the metabolic pulse-chase data suggests that receptor cleavage is a rapid and efficient process. Moreover, our results indicate that the receptor N-terminus is released from the cells by shedding, a phenomenon rarely described for GPCRs. Immunofluorescence microscopy with subcellular markers indicates that GPR37 is still in the full-length form in the trans-Golgi network but is predominantly expressed in the cleaved form at the cell surface. Additionally, experiments with various proteinase inhibitors imply that the receptor is cleaved by a metalloproteinase. As proteolytic processing is involved in the regulation of many cell surface receptors, our findings provide valuable information about GPR37 that help to understand its function and role in AR-JP at the molecular level.
